# An iterative topic model filtering framework for short and noisy user-generated data: analyzing conspiracy theories on twitter

**DOI:** 10.1007/s41060-022-00321-4

**Published:** 2022-05-06

**Authors:** Gillian Kant, Levin Wiebelt, Christoph Weisser, Krisztina Kis-Katos, Mattias Luber, Benjamin Säfken

**Affiliations:** 1 University of Göttingen, Göttingen, Germany; 2 Campus-Institut Data Science (CIDAS), University of Göttingen, Göttingen, Germany

**Keywords:** Latent Dirichlet allocation, LDA, Conspiracy theories, Iterative filtering, Hashtag pooling, SARS-CoV-2, Covid-19, Sentiment analysis, Geo-spatial analysis, NLP, NLP pre-processing

## Abstract

Conspiracy theories have seen a rise in popularity in recent years. Spreading quickly through social media, their disruptive effect can lead to a biased public view on policy decisions and events. We present a novel approach for LDA-pre-processing called Iterative Filtering to study such phenomena based on Twitter data. In combination with Hashtag Pooling as an additional pre-processing step, we are able to achieve a coherent framing of the discussion and topics of interest, despite of the inherent noisiness and sparseness of Twitter data. Our novel approach enables researchers to gain detailed insights into discourses of interest on Twitter, allowing them to identify tweets iteratively that are related to an investigated topic of interest. As an application, we study the dynamics of conspiracy-related topics on US Twitter during the last four months of 2020, which were dominated by the US-Presidential Elections and Covid-19. We monitor the public discourse in the USA with geo-spatial Twitter data to identify conspiracy-related contents by estimating Latent Dirichlet Allocation (LDA) Topic Models. We find that in this period, usual conspiracy-related topics played a marginal role in comparison with dominating topics, such as the US-Presidential Elections or the general discussions about Covid-19. The main conspiracy theories in this period were the ones linked to “Election Fraud” and the “Covid-19-hoax.” Conspiracy-related keywords tended to appear together with Trump-related words and words related to his presidential campaign.

## Introduction

The year 2020 was dominated by the Covid-19 pandemic. Facing the pandemic demands a competent administration and public support for its decisions. However, public decisions are taken under substantial uncertainty: knowledge about the virus and scientific recommendations to stop the spread of the Covid-19 disease are constantly being updated. Previous policy recommendations may become refuted by new insights and developments. This may lead to an erosion of confidence in scientific and political authorities. All these developments are a fertile ground for conspiracy theories to thrive and disrupt the public discourse [4]. Public discourse is a key component of the political decision-making process [24]. Conspiracy theories disrupt this process by misinforming public opinion [11]. Therefore, it is important for policy makers and journalists alike to obtain reliable information about how and when misinformation spreads on social media platforms.

We investigate the public discourse on Twitter which has become a major tool of information acquisition in the recent years [2]. Despite its large popularity, Twitter text data is highly unstructured and it can be challenging for researchers to generate meaningful topics from their sampled data. We estimate Latent Dirichlet Allocation (LDA) Topic Models [5], embedded in a pre-processing framework that enables us to better identify conspiracy-related posts. For this purpose, we present a novel approach for LDA pre-processing that we call Iterative Filtering in combination with Hashtag Pooling. With these techniques, we are able to frame the discussion and to identify relevant topics, regardless of the inherent noisiness and sparseness of tweets. We apply these techniques to gain new insights into the temporal development of conspiracy-related topics on Twitter during the Covid-19 pandemic. We rely on data from the last four months of 2020, a time period in which public discourse has been dominated by US-presidential elections and allegations of electoral fraud. Additionally, we also analyze the polarization of conspiracy-related tweets in terms of their spatial distribution and sentiment. We find that the main conspiracy theories over this time period were related to “Election Fraud” in November and December and the “Covid-19-hoax” theory in general. Thereby, conspiracy-related keywords tend to appear together with Trump-related words and words related to his presidential campaign.

Our Iterative Filtering framework allows us to descriptively explore which conspiracy theories exist on Twitter and how much attention they generate, either by their proponents, or their opponents. However, our framework cannot clearly identify misinformation attempts, only the public engagement with these phenomena.

The paper is structured as follows. Sect. 2 discusses the related literature. Our method is presented in Sect. 3. Section 4 describes the data and implementation of our approach. In Sect. 5, we present the results of the topic model estimations, and illustrate the geo-spatial spread of various conspiracy theories together with a spatial distribution of sentiments. Finally, we conclude our findings in Sect. 6.

## Related literature

Analyzing topical content on Twitter has been the focus of numerous studies, many of which combine LDA and Sentiment Analysis to analyze Twitter Data and text data in general. Our proposed approach has the potential to improve such analyses in the future. Our work is especially closely related to the literature that uses different pre-processing approaches (see, e.g., [1]). Our contribution gives researchers a tool to improve resulting LDA topics in terms of their coherence when fed with short and sparse text such as Twitter data.

Several applications focus on analyzing Twitter data by LDA Topic Modeling to answer questions of socio-political significance [6, 17, 32]. [17] analyze the sentiments of news articles and Twitter data during the Ebola outbreak in 2016. By feeding a LDA algorithm with both tweets and news articles, they are able to model the topical and sentiment changes over time. In a similar vein, [6] present a temporal LDA-based analysis on Covid-19-related Twitter activity. [32] employ LDA-derived latent topics for Sentiment Analysis on the topic of Covid-19. They filter their data by a few Covid-19-related keywords beforehand.

However, the topics produced by the models mentioned above seem to lack topical coherence and distinguishability in most cases, even though some models were fed with between two million [32] and over seven million tweets [17]. A reason for this could be that all three publications define a tweet as a single document for LDA [22]. In our presented framework, Hashtag Pooling is a vital pre-processing step that allows us to avoid this problem.

Our use case and technical analysis of Twitter data is closely related to the approach proposed by [14]. Their study offers a natural comparison case for our approach. [14] analyze sentiment on Twitter on a temporal and spatial level. Sentiments are plotted on US-county level and mapped over time. An eight-topic-LDA model is trained on the whole corpus of tweets. In contrast to [14] who preprocess their data to remove any bots form their corpus, we do not remove tweets that are presumably generated by bots in our approach. Bots may play an important role in shaping the public discourse by setting a minority’s opinion on the agenda. Additionally, [14] do not apply Iterative Filtering and Hashtag Pooling as we suggest in this paper. As a consequence, their approach results in the creation of rather generic topics like “Enjoy the weekend” or “Great time on Sunday.” By contrast, our Iterative Filtering approach filters the public discussion by zooming in on targeted themes with every iteration-step. As a result, we are able to feed the LDA model with a collection of tweets that are much more coherent and therefore more insightful. The case we present in this paper is a good example of how Iterative Filtering can improve LDA results on short and sparse data.

In a further subset of the literature, filtering the dataset for Covid-19 related tweets becomes a crucial step. [1] use LDA Topic Modeling to differentiate between informative and uninformative tweets concerning the Covid-19 discussion and identify trending topics on Twitter. [1] preprocess their dataset by training a classifier to exclude irrelevant tweets before performing topic modeling. One of the main downsides of this approach is the necessity of labeled data to establish the relevance of a tweet. In contrast to our Iterative Filtering approach, using a classifier to filter tweets crucially relies on the presence of labels in the training data that allow to identify the topics of interest. Using Iterative Filtering, only a handful of keywords are needed that allow us to target the topics of interest, while further keywords will be automatically generated in the process of filtering.^1^

As mentioned before, the literature on the topics of misinformation detection on social media using topic modeling and sentiment analysis is very diverse. Further useful information can be found in [10] (utilize LDA as a descriptive tool), [31] (Sentiment Mixture Model), [20] (Twitter Opinion Topic Model), [33] (LDA-based Sentiment Analysis) and [25] (Network Graph Analysis).

In summary, our approach focuses especially on data pre-processing as a crucial step to substantially increase LDA topic coherence and introduces a framework that can be used to gain deeper insights into targeted topics in noisy user-generated content. Hence, our framework can serve as a basic analytical step preceding a deeper analysis for researchers interested in certain topic-driven discussions on social media.

## Methods

### LDA topic models

An LDA is a generative probabilistic model. We define the corpus as a collection of *M* documents, $\mathbf{D} = \{\mathbf{w }_{1},...,\mathbf{w }_{M}\}$. A sequence of words establishes a document, $\mathbf{w} = (w_{1},...,w_{N})$. As formulated in [5], words are representations of unit-basis vectors and are indexed on a fixed vocabulary in $\{1,...,V\}$. These words $w_n,\ n = 1,\ldots ,N$ are affiliated with a document’s unobserved topic variables $z_n,\ n = 1,\ldots ,N$. Therefore, the joint probability distribution is defined as $p(\varvec{\theta }, \mathbf{z} , \mathbf{w} | \varvec{\alpha }, \varvec{\beta })$ of $\varvec{\theta }$, $\mathbf{z}$ and $\mathbf{w}$ as

1 $$\begin{aligned} \qquad \qquad p(\varvec{\theta } | \varvec{\alpha }) \prod _{n=1}^{N} p(z_{n} | \varvec{\theta }) p(w_{n} | z_{n}, \varvec{\beta }) \end{aligned}$$

where $\varvec{\alpha }$ are the corpus-wide parameters of a Dirichlet distribution, $\varvec{\beta }$ the corpus-wide word probability matrix, and $\varvec{\theta } \sim Dir\left( \varvec{\alpha }\right)$ the document-level *k*-dimensional topic mixture. On this basis, the marginal distribution of a document $p(\mathbf{w} |\varvec{\alpha }, \varvec{\beta })$ and the probability of a corpus $p(\mathbf{D} |\varvec{\alpha }, \varvec{\beta })$ can be formulated as in [5]. With the inference we obtain the corpus wide parameters $\varvec{\beta }$ and $\varvec{\alpha }$ as well as $\varvec{\theta }_j,\ j=1,\ldots ,M$, that can be interpreted as the document-specific probabilities of *k* topics.

While there are different approaches for inference, we apply the Online Variational Inference for LDA algorithm by [12] that uses a variational inference approach. Its benefits lie in an optimized memory usage and computation time.

The inference problem is the untraceable posterior distribution. Variational approaches therefore approximate the untraceable posterior by other traceable distributions. For the approximation of the true posterior, the Kullback–Leibler divergence is used to minimize the distance between two probability distributions [5]. Finally, the parameters of the variational posterior are approximated by the Expectation Maximization algorithm. The algorithm of [12] is implemented in the Python^2^ package Gensim,^3^ which we use in this paper. A different option to a variational implementation is a Markov-Chain-Monte-Carlo approach using Gibbs Sampling [7].

### Iterative filtering

**Fig. 1:**
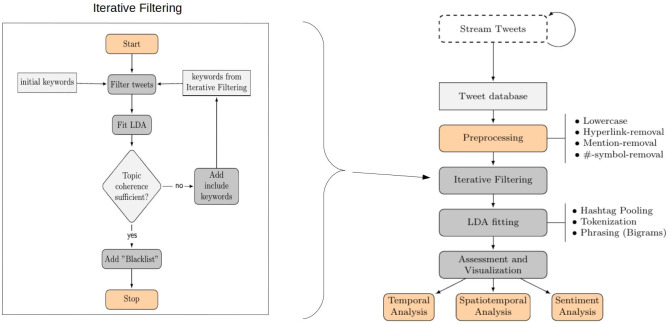
Iterative Filtering process (left) and Pipeline (right)

Two main issues when dealing with Twitter data are their sparseness and noisiness. Tweets are short texts (of maximum 280 characters)^4^ and use a lot of informal language and slang. LDA Topic Models normally do not perform well on short documents [13]. As shown in Sect. 2, most studies ignore this problem and estimate a LDA model where each tweet is used as a single document. This may lead to generic and incoherent topics. As a remedy, we introduce a combination of two approaches: Hashtag Pooling and Iterative Filtering. The idea of the Hashtag Pooling was introduced by [22]. [22] developed a Hashtag Pooling algorithm, which concatenates the textual contents of tweets that use the same hashtag. The hashtags themselves are part of the text and are therefore also included in the concatenated tweet. [22] show that tweets that are concatenated by a hashtag to form longer pseudo-documents improve the coherence of LDA topics significantly. Additionally, as proposed by [22], we measure the cosine similarity between the generated pseudo-documents and single tweets that carry no hashtag. If a predefined similarity threshold is passed, the single tweet is attached to the matching pseudo-document. We use a self-implemented version of their proposed algorithm in this paper.

Before applying Hashtag Pooling on the text data, we implement conventional preprocessing steps. These include the removal of links, mentions, symbols and stop words. Additionally, all tokens were preprocessed as bigrams. These steps are well established and are standardized measures of data preparation [30]. The full order of the process can be seen in Figure 1.

Furthermore, we introduce a novel technique that we call Iterative Filtering. When analyzing Twitter data for a specific parent topic like “Conspiracy Theories,” we need an effective filter technique to remove unrelated tweets and at the same time include as many related tweets as possible. We start with defining a parent topic of interested, which is the discussion about conspiracy theories in the application of this paper. To do this, we search manually for relevant keywords in samples of Twitter data that are related to the parent topic. This results in a small dictionary of 11 filter terms: *qanon, wwg1wga, pizzagate, chemtrails, earthisflat, lizardpeople, newworldorder, covid19, virushoax, trumprussia*, as well as *fakenews*. This choice aims to capture the “classical” conspiracy theories [9] like flat earth or chem trails, as well as more recent phenomena like Q-Anon or the term “Fake News,” plus Covid-19-related terms. The specification of keywords is also a means for users to use their knowledge about the topic that they aim to explore on Twitter. In our case, for example, prior knowledge about some of more recent conspiracy theories that are linked to the elections is incorporated. This initial definition of the keyword set is to a large extent subjective and can be considered as a prior of keywords that define the parent topic. Filtering our tweet database with only a few keywords results in few tweets remaining for LDA analysis.

To increase our list of filter terms and thereby include more tweets into our analysis, we perform Iterative Filtering. For this we fit an LDA prematurely (after applying the pre-processing steps described above) to the dataset filtered with our initial few keywords. We explore the resulting topics for “new” words that serve as good filter terms and add them to the filter dictionary. The decision on which words to add is based on a subjective evaluation of the words’ importance and connection to the targeted theme (i.e., conspiracy theories). We filter our original tweet database again with the increased filtering dictionary and thereby obtain a larger dataset of filtered tweets, on which we again perform a premature LDA analysis to find new filter keywords. Note that the filtering is conducted on the unpooled (original) tweets. The pooling is performed as part of the pre-processing of the LDA estimation in each iteration.

The three steps of filtering the database, fitting an LDA, and exploring and adding new filter terms are repeated until no new and relevant filter terms appear in the resulting estimated topics. Note that in every iteration round the same initial dataset is used to apply the filter based on the updated dictionary of keywords.

In a last step, we build a blacklist-dictionary to identify uninformative words in smaller topics of the LDA. For the final estimation round, we remove all tweets that contain uninformative words, defined in the blacklist dictionary. By this, we filter out those tweets that are uninformative even though they contain words from the Iterative Filtering steps, for instance, because they are using the keywords in a different context as the targeted themes or they are occurring temporally manner. An example are tweets that use the blacklist keyword *nye*, which stands for “new years eve.” This word occurred in some of the relevant topics in a few estimations, due to an increased usage around the end of 2020. We therefore put it on the blacklist due to its uninformative nature for our targeted topics. The blacklist is therefore essentially a means to clean the topics and to increase in the topics’ coherence. The interpretation of topics by humans is the subjective part in topic modeling. Similarly, the selection of keywords from the estimated topics as part of the updating step of the filter dictionary requires human interpretation and is therefore subjective. However, the benefit of this subjective element in our framework is that it allows the estimation of very specific topics. The algorithm is visualized on the left side of Figure  1.

In our overall pipeline (on the right side of Fig. 1), most steps are automated. The only parts that demand manual user input are parts of the Iterative Filtering process. These include the choice of initial keywords, the choice of the number of topics for the LDA estimation, the evaluation of the results to find new keywords and adding them to the filter dictionary, and the choice of the “Blacklist” keywords. The number of topics must be chosen subjectively to ensure a better interpretability of the results. The number of topics might be adjusted by the user during the Iterative Filtering process to improve the coherence of the resulting topics and the amount of tweets that are part of the dataset to be analyzed. As a rule of thumb, the number of topics should be decreased if the amount of “residual topics,” which are topics that are inherently fuzzy, incoherent and do not provide any insights into the targeted topics (here: “Conspiracies” and “Covid”), comprises around half of the topics of an LDA. The number of topics should be increased if singular topics subjectively carry more than one coherent topic in themselves.

The number of starting filter keywords is chosen arbitrarily and is dependent on the users’ prior knowledge about relevant words in the topic of interest. A potential issue arises if the user chooses low quality keywords at the beginning or during the process. This would lead to a lower coherence of the topics. This is especially challenging when some words or hashtags have a meaning that is unclear on first sight. A good example would be the hashtag “savethechildren.” It is a phrase that was taken over, reframed in its meaning and became widely used among conspiracy theorists. The best way to find out more about the context of a specific term is to use an internet search engine to search for results that set the term in connection to the topic of interest (here: “savethechildren” plus “conspiracy”) or to look up some tweets that use this specific term to get a feeling of the context it is used in. Rather than relying on a pre-specified threshold, we prefer to choose the words from the overall context of the topic of interest. For a tweet to become part of the data to analyze, a word that has been chosen in the Iterative Filtering process just has to occur once in a tweet. This tweet is then added to the dataset. This approach obviously does neglect tweets where typos in the keywords occur. Additionally, regarding the robustness of our approach, we want to point out the path-dependency of the framework: The subjective choice of which keywords are added to the filtering list determines the inclusion of the tweets in every following iteration. Therefore, it is important that the users of our framework critically reflect on keyword selection in every iteration step. Our approach produces a biased sample of the main dataset to identify highly specific and coherent topics of interest. These topics are not identifiable in the full dataset, because the LDA model is not able to discriminate between marginal topics when no filtering is applied.

**Fig. 2:**
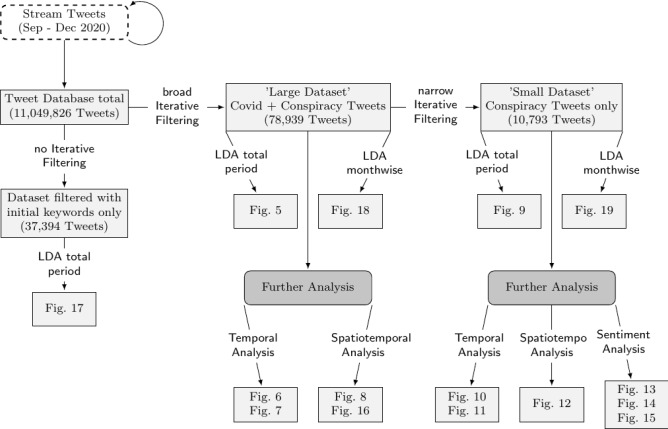
Overview of datasets and plots. In “Broad Iterative Filtering,” a relatively large set of keywords is used for the filtering. “Narrow Iterative Filtering” indicates that a subset of the keywords from the initial broad Iterative Filtering is used to find very specific topics

To show the capabilities and effectiveness of our Iterative Filtering framework, we included an example of the results of the LDA topic distributions, on which we only have used the initially selected conspiracy- and Covid-related filter keywords that were mentioned above. Figure  17 in the Appendix shows the topic distribution of a dataset without Iterative Filtering. The differently sized circles (or “bubbles”) represent LDA topics, while their size is reflecting the number of total tokens from the corpus that are being used by the specific topics (excluding tokens with an inner-topic prevalence of zero). The visual representation itself relies on a principal component analysis (PCA) of the topic distributions and the distances between the topic bubbles are calculated by the Jenson–Shannon distance measure, which compares the similarity of probability distributions [19]. The words shown on the right side of the plot are the most important words for a selected topic. In our plots, we sometimes adjusted the $\lambda$ parameter of the relevance metric to better present rare, but topic-defining words. These rare words are ranked high when a small $\lambda$ value is selected. A low $\lambda$ gives more weight to a terms lift, which is defined as ”the ratio of a term’s probability within a topic to its marginal probability across the corpus” [26]. By contrast, a larger $\lambda$ increases the weight of the topic-specific probability of a word in the ranking. Details on the metric can be found in [26]. Without using Iterative Filtering, the most frequent topic type consists of “Residual topics.” They account for about 20% of all tokens in this analysis and over half of the number of topics in total. There are very generic Covid topics present, but the rather small conspiracy topics almost completely disappear among the large amounts of unrelated noisy tweets. The LDA fit does not represent them. Additionally, by filtering only by the initial keywords, this analysis only includes 37,394 tweets, which are less than half of the “large dataset” in Fig. 2, which contains 78,939 tweets when filtered with all keywords obtained by the Iterative Filtering process. This example shows that applying the Iterative Filtering framework increases the amount of relevant data and allows us to “zoom in” to smaller discussions inside our tweet database. We invite the reader to compare this baseline model to the other results that are presented in the following sections to see the improvements achieved by Iterative Filtering.

### Sentiment analysis

For Sentiment Analysis, we use the rule-based tool VADER [15], which works well for noisy texts. Such rule-based methods assign sentiment scores to single terms in a text and aggregate those to attain an overall sentiment. VADER computes scores for the categories negative, neutral and positive which are normalized such that they sum up to one. Especially for tweets, VADER outperforms more elaborate Machine Learning procedures, such as support vector machines or naive Bayes classifiers with accuracy scores of up to 96% [15].

## Data and implementation

The data collection and implementation of our analysis are visualized on the right side of Fig. 1. The data collection has been realized via the Twitter API using the Python package TTLocVis [16]. The Twitter API permits the streaming of current Twitter content in real time, which we restricted to the US-mainland area. The data collected using the API is a randomly sampled dataset of all tweets published during streaming. Its size is limited by the APIs rate limit.^5^ In our analysis, we used streamed US tweets from September to December 2020. On this data, we implemented the Hashtag Pooling procedure as described in Sect. 3.2.

**Fig. 3:**
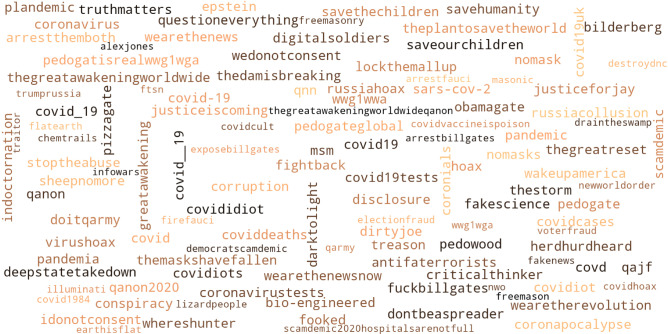
Keyword dictionary after iterative filtering

**Fig. 4:**
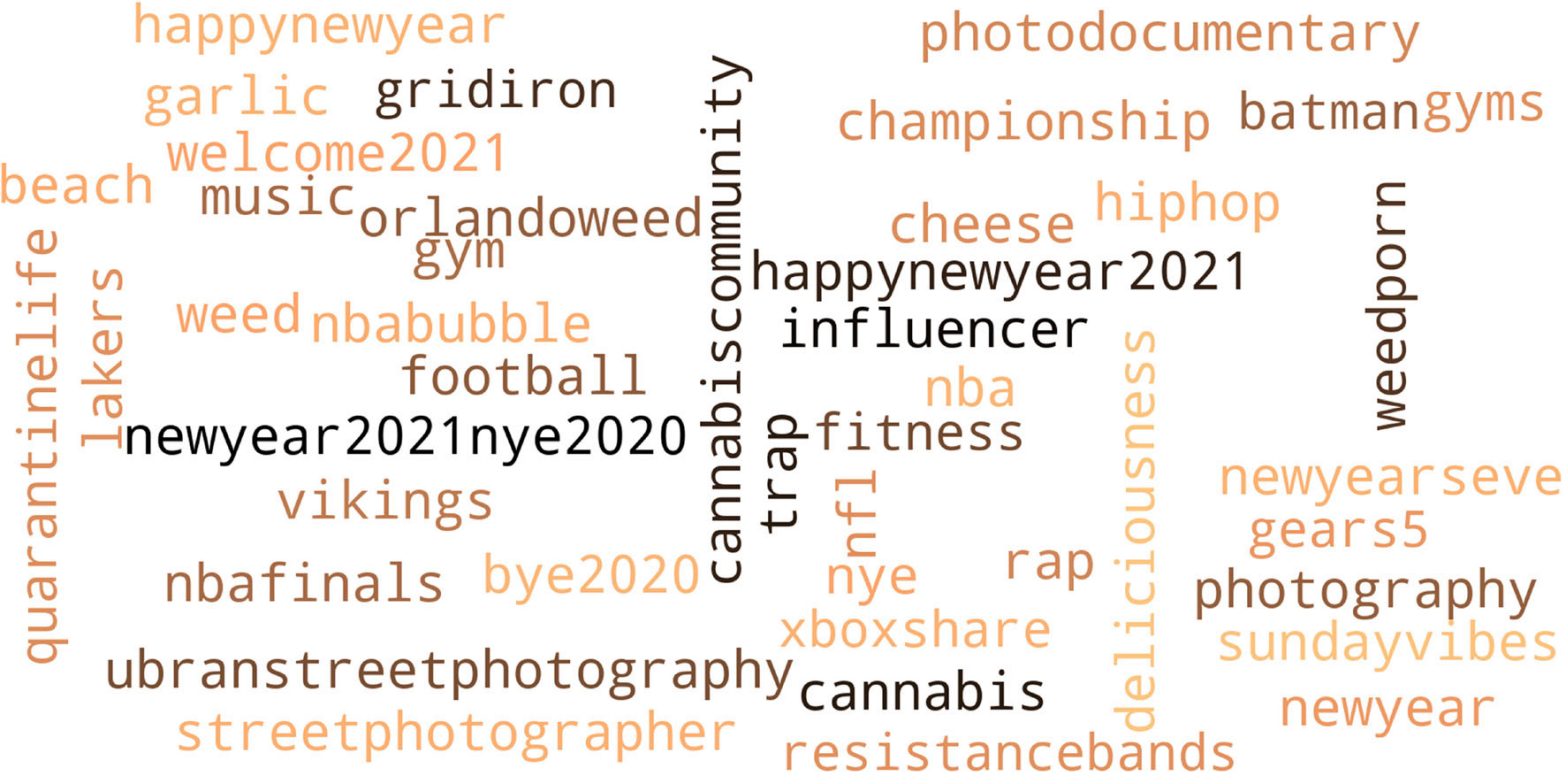
Blacklist dictionary

The final dictionaries can be found in Figs. 3 and 4 . The former contains the included keywords after Iterative Filtering, while the latter displays our blacklist dictionary. After Iterative Filtering, we copy all conspiracy-related terms and create a new dictionary to produce two datasets. The first contains Covid-19 and conspiracy-related words, resulting in a dataset of 78,939 tweets. The second contains conspiracy-related keywords only, resulting in a smaller dataset of 10,793 tweets, a sub-set of the first. This is visualized in Fig. 2. The large dataset is dominated by discussions about Covid-19 in general and conspiracies in the context of Covid-19, while the small dataset allows us to dive deeper into controversial discussions related to conspiracy theories.

For each of the two datasets, a twenty-topic LDA model is estimated, covering the whole time period from September to December 2020. Additionally, grouping the tweets by month, we estimate four eight-topic LDA models for the larger, and four ten-topic LDA models for the smaller dataset. This results in a total of ten LDA Topic Models. The visualizations are implemented using the Python package PyLDAvis [26].

Based on these ten models, we conduct an analysis on the spatial and sentiment level to find out more about the geographic distribution and sentiment content of conspiracy theory-related tweets in the USA. We hope to find indications that tweets with a high prevalence of conspiracy-related topics differ from non-conspiracy-related topics sentiment-wise.

For the Sentiment Analysis, we consider the scores for the positive and negative sentiment categories and select tweets with sentiment scores that are larger than zero. By that we exclude tweets that receive a score value of one in the neutral category. The sentiment visualizations are based on the sentiment score. For example, if a tweet has a negative score of 0.7 and positive score of 0.3, both sentiment scores are visualized in the respective graphs for positive and negative sentiment.

As a final note, [8] point out, that likelihood-based topic model evaluation does not always correspond to topics that seem semantically meaningful to human interpretation. The same issue arises when automatically labeling LDA-generated topics. Therefore, we evaluate the LDA topics by human annotation.

## Results

The Results Section is structured as follows. In Subsection 5.1, we describe month-wise estimates of the large Covid-19-tweets dataset, followed by month-wise estimates of the small conspiracy-tweets dataset in Sect. 5.2. Afterward, we focus on spatiotemporal characteristics by describing estimates of the total sample period of September to December 2020. In Sect. 5.3, we present the results of an LDA model with twenty topics on the large Covid-19-tweets dataset and subsequently on the small conspiracy-tweets dataset in Sect. 5.4. Finally, sentiments are discussed in Sect. 5.5.

### Topic modeling: conspiracy theories in the context of Covid-19

The plots in Fig. 18 in the Appendix show the annotated topics for estimates on monthly data from September to December 2020. The plot for September (upper left of Fig. 18 in the Appendix) shows that topic 4 (19.3% of all tokens) and topic 7 (3%) contain conspiracy-related keywords. Topic 4 is mainly discussing political activism and the upcoming US-presidential election. In comparison, topic 7 seems to go in a similar direction but appears to be more controversial than topic 4: The conspiracy-related keywords are more aggressive (*traitortrump_trumptreason, obamagate*) and, in general, are more critical toward Trump at the first sight. The tweets also directly target the president, accusing him of treason for example. The Covid-19-related topics dominate the results. Topic 5 (39.1%) is the main topic discussing the pandemic and is also the largest one in terms of the number of tokens for this LDA model. It contains almost exclusively the most important Covid-19-related keywords like *pandemic, cases, healthcare* and *deaths*. Topic 2 (17.4%) is another Covid-19-related topic, where people discuss social distancing, promote wearing masks, and advise on how to cope with the pandemic. The model also produces residual topics like topics number 3, 6 and 8 (cumulative 11.1% of all tokens). Nevertheless, in comparison with the results of the LDA analysis in Fig. 17 in the Appendix, only a small percentage of tokens is now uninformative. Despite the improvement in coherence of the topics due to Iterative Filtering, Twitter data remains inherently noisy if (semi-)automated measures for pre-processing are used. Hence, the residual topics help to capture remaining uninformative tweets.

The LDA model covering the tweets for October (upper right of Fig. 18 in the Appendix) is strongly shaped by the upcoming election. Although the pandemic topic remains still the largest (with 30.9%, topic 1), different aspects of the US-Presidential Election are captured in the topics 2 (12.4%), 3 (26.4%) and 4 (7.2%). Conspiracy-related keywords can mainly be found in topic 4 (*fakenews, msm, whereshunter*) which is a Trump-supportive topic. Topic 3 is intersecting topic 2 and topic 1 by building a “bridge” between the Covid-19 pandemic and the election. It mainly covers the event of Donald Trump being infected with Covid-19. We find no conspiracy-related keywords among the top words (except for *hoax* in topic 3) for the topics of interest.

In November (lower left of Fig. 18 in the Appendix), we see a large amount of conspiracy theories, flooding especially the discussion about the US-presidential election. Around 25% of the volume of this dataset is shaped by the discussion about election fraud (topic 2, 4.8% and topic 7, 22.6%). Topic 7 contains tweets discussing the results of the election and comes with conspiracy keywords like *electionfraud, voterfraud* and *fakenews*. This indicates that conspiracy theories with regard to the election were strongly prevalent in the public discourse. Independent investigations have shown no evidence of voter fraud in the US-Presidential Election of 2020 of significant scale [21] and no increases of election fraud due to vote-by-mail [3]. Topic 2 includes aggressive sentiments, calling for protest and resistance against the assumed “voter fraud.” The topic is almost exclusively constituted of conspiracy-related keywords. Another noteworthy development is that a “vaccine” topic can be observed (topic 5, 4.2%) which is distinct from the main pandemic topic (topic 6, 30.5%). Here, we can see that our approach is able to describe important new developments on the targeted topics if they are not only marginally discussed.

In December (lower right of Fig. 18 in the Appendix), we can see a shift in the debate about Covid-19: The main Covid-19 topic morphs into a “Covid vaccine” topic and additionally grows in size (topic 5, 47.7%). During this month, the public discourse about Covid-19 flared up once more around the issue of vaccines. We find no conspiracy-related keywords in all Covid-19-related topics. Vaccination-related conspiracies seem to have been marginalized in this period. Also in December, the amount of conspiracy-related tokens decreases. Topic 7, related to “Election Fraud” (with 10.7%), shows that the discussion about this specific conspiracy theory was decreasing strongly compared to the previous month. The tone, nevertheless, stays aggressive, containing key words like *bidencrimefamily, bidencheated, treason* and *stopthesteal*.

To conclude, our results show that conspiracies about big political events that have recently occurred are likely to penetrate the public discourse. During this same time, Covid-19-related and other conspiracy theories became rather marginalized in the main discourse. In the next part, we will have a closer look at the prevalence and distribution of conspiracy theories by analyzing LDA models that have been iteratively filtered on conspiracy-related keywords only.

### Topic modeling: zooming into the distribution of conspiracy theories

In this section, we discuss results produced by LDAs fed with monthly data, which have been filtered only by keywords with regard to conspiracy theories. This small dataset is a subset of the larger Covid-19-tweets dataset in Sect. 5.1. While the dataset from the Section above consisted of 78,939 tweets in total, the data in this section only contains 10,793 tweets. The results can be found in Fig. 19 in the Appendix.

In September (upper left of Fig. 19 in the Appendix), topic 4 (17.6% of all tokens) contains a polarized discussion about child abuse, probably amplified by the release by the controversial Netflix movie “Cuties” during that month [23]. We find a few conspiracy-related keywords in this topic like *pedogate2020* and *pizzagate*. Topics 3 (12.1%) and 10 (16.7%) are large conspiracy-related topics, discussing election fraud already well before the election, and also contain media critique in form of a “Fake News” discussion. Two other very interesting topics are topic 5 (5.3%) and 7 (7.9%). Topic 5 is a Q-Anon related topic, discussing “The Great Awakening,” while topic 7 perfectly represents the discussion about Covid-19 as planned or non-existing, also influenced by the Q-Anon group. As seen in the previous section, we find that the “Plandemic” conspiracy theory is rather marginalized in relation to all Covid-19 related discussions, but plays an important role in the context of all conspiracy-related topics. Finally, topic 8 (1.7%) shows that other well-known “classical” conspiracy theories like the Illuminati play a marginal role within the context of conspiracy theories in this time period.

For October (upper right of Fig. 19 in the Appendix), we find a similar composition of topics as in September, but with a different distribution. The “Fake News” topic 10 (11.4%) is still prevalent, but has shrunk in size. Also, a small anti-vaccine topic has developed (topic 8, 3.2%) while the “Plandemic” topic (topic 6, 25.8%) merged with a Trump-related discussion. The reason may be, as already stated in Sect. 5.1, that in October 2020 Trump was hospitalized due to an infection with Covid-19. Also, the upcoming election casts its shadow ahead as we find a “Pro-Trump” topic (topic 2, 18.1%) and a growing “Election Fraud” topic (topic 4, 13.8%). Additionally, we also find a rather marginalized “Obamagate” topic (topic 1, 1.4%) and a topic accusing the Biden family of crimes and corruption (topic 5, 10.2%). These examples reveal that some conspiracy theories focus on public figures to discredit their actions as well as their person as an illegitimate political opponent.

In November (lower left of Fig. 19 in the Appendix), the US-presidential election dominates all discussions, so that a ten-topic-LDA does not produce any insightful results anymore. Therefore, we aggregate topics by estimating a five-topic LDA for this month. We find that “Election Fraud” (topic 3, 38.6%) and “Media Criticism” (topic 5, 33.4%) have the highest prevalence. The most interesting topic is perhaps topic 1 (16.2%), calling for resistance against the election results. Misinformation like this can indeed lead to serious damage of democratic institutions [18] and even real-world violence. This was for instance evident during the storm of the Capitol in Washington D.C. on the 6th of January 2021. Here, we can find the first hints about public invocation (keywords *marchfortrump_millionsmagamarch* and *fightback*) that led to scenes like the ones seen at the Capitol a few weeks later. We find a small “New World Order” topic (topic 4, 6.5%), another hint that “classical” conspiracy theories play a marginal role in this time.

Finally, in December (lower right of Fig. 19 in the Appendix), we observe a decrease in the election-related discussion, although, the main topics still refer to elections. Topic 3 (13.2%) discusses ballots and court decisions in relation to the election, while topic 4 (24.5%) discusses the role of the media in election fraud. Topic 8 (8.3%) is a media-related topic built around the website *Infowars* and discusses mainly election fraud in an aggressive tone. Additionally, we find a rather small Trump and GOP critical topic (topic 9, 4.7%) and a much larger topic discussing resistance to election fraud (topic 5, 29%). All accumulated, we still have over 80% election related conspiracy topics in December. Nevertheless, there are two more topics noteworthy, a topic about Covid-19 being a planned pandemic as we also have seen it in November (topic 7, 6.3%) and a very small topic that describes the vaccine as part of the plan for a “New World Order” (topic 2, 2.1%), building upon a “classical” conspiracy theory.

There are two main conclusions from these findings. First, the dominating theory by far is “Election Fraud,” followed by the “Plandemic”/“Covid-19-hoax” conspiracies. Secondly, “classical” conspiracy myths like lizard people, chem-trails, flat-earthers, Illuminati or “New World Order” are marginalized.

Our narrative evidence suggests that the potential of conspiracy theories to spread within the public discourse is especially large whenever they refer to already controversial topics that are in the focus of the public attention. Successful conspiracy theories seem therefore to adapt to the zeitgeist to leverage the popularity of a topic in order to attract new followers.

### Topic modeling: spatiotemporal characteristics of the Covid-19 discussion

This section investigates the spatiotemporal components of a twenty-topic LDA estimated on the large Covid-19-tweets dataset over the whole sample period. We cover the characteristics of the month-wise eight-topic models from Sect. 5.1 as well. In order to achieve this, we increased the topic numbers from 8 to 20 topics.

**Fig. 5:**
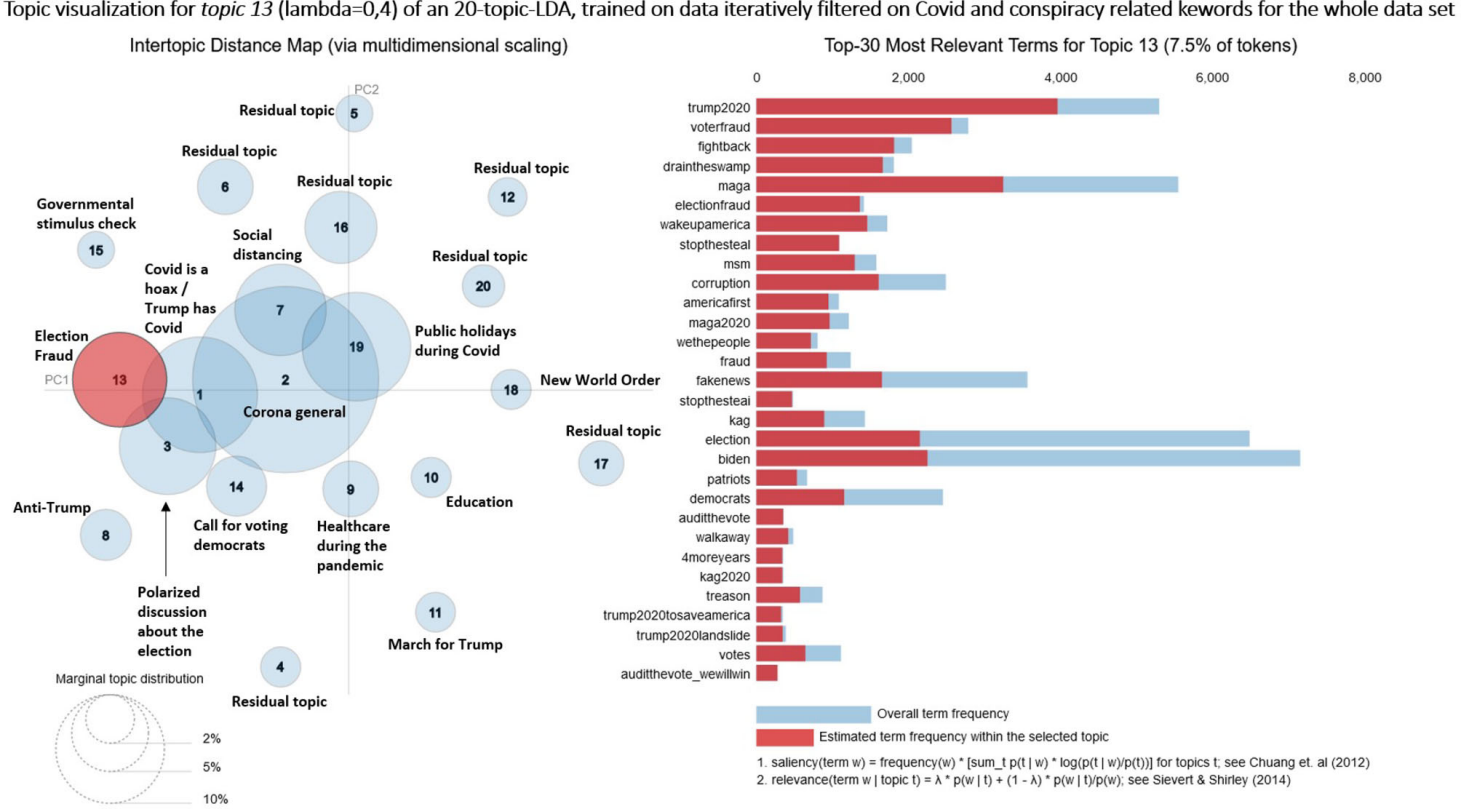
LDA visualization of total time period—Covid-19 and conspiracy-related tweets

The inter-topic distance map (Fig. 5) indicates a good LDA performance: The residual topics are small and located far away from the topics of interest in the center of the plot. The majority of corpus terms are allocated to positive and supportive topics, such as public holidays during the pandemic or social distancing. About 40% of all terms belong to these general topics. In contrast, around 10% of all terms are allocated to the conspiracy topics 11, 13 and 15, which cover concerns about election fraud and support for Trump.

**Fig. 6:**
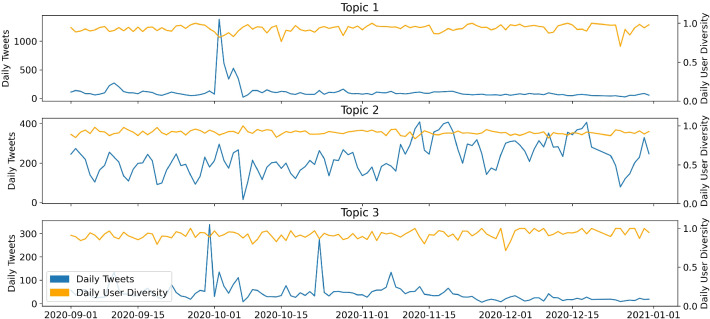
Time series of Covid-19-conspiracy-LDA topics 1, 2, 3

**Fig. 7:**
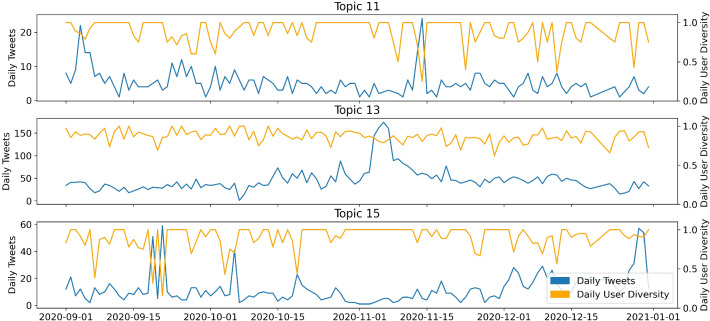
Time series of Covid-19-conspiracy-LDA topics 11, 13, 15

Figures 6 and 7 show the temporal dimension of the estimated topics. Each tweet in the dataset is assigned to the topic with the highest estimated topical prevalence. The left axis shows the number of tweets per day. The right axis shows the ratio of the number of tweets relative to the number of different users for a specific time point, capturing user diversity of a topic. A low user diversity indicates that few accounts dominate the discussion of a topic, thus few users are very influential. The user diversity measure is very sensitive if daily sample sizes are low for the respective topic. In this case, the graph displays high volatility.

Looking at the time series (Figure 6), it is noticeable that some topics are assigned tweets from the entire sample period, while others are exclusively assigned tweets for a time slot of a few days. Hence, we can distinguish between long-term topics, being discussed for longer periods of time, and short-term topics, which disappear quickly. Topic 1 (11.2% of all tokens) is such a short-term topic and peaks in the second half of October. It discusses Donald Trump’s Covid-19 infection. In contrast, topic 2 (29.3%) is a long-term topic that covers many aspects of the pandemic, such as day-to-day pandemic activities or quarantine measures. After the announcement of the vaccine by BioNTech-Pfizer on November 9th, there is an increase in daily tweets for this particular topic. Topic 3 (7.9%) is again a short-term topic, but with several peaks. Those are reactions to the presidential debates on September 29th and October 15th. For all three topics, the user diversity increases to 0.75. There are no noticeable drops, except during days when sample sizes are low for the short-term topics 1 and 3. Topic 11 represents the “classical” conspiracy theory “Illuminati.” It is small with a maximum of 20 tweets per day and is mostly discussed in September and mid-November. During the remaining sample period, only a few tweets are assigned to this topic. Topic 13 (7.5%) concerns the election fraud myth and displays a lower user diversity on average. This may indicate that an active subgroup of users is driving the majority of the discussion. Especially during the peak around the election, we observe a drop in user diversity. This could also be a hint of an involvement of bots in this topic. The drop in December appears more drastic; however, sample sizes are lower in this period. This leads to a less robust user-diversity-measure.

**Fig. 8:**
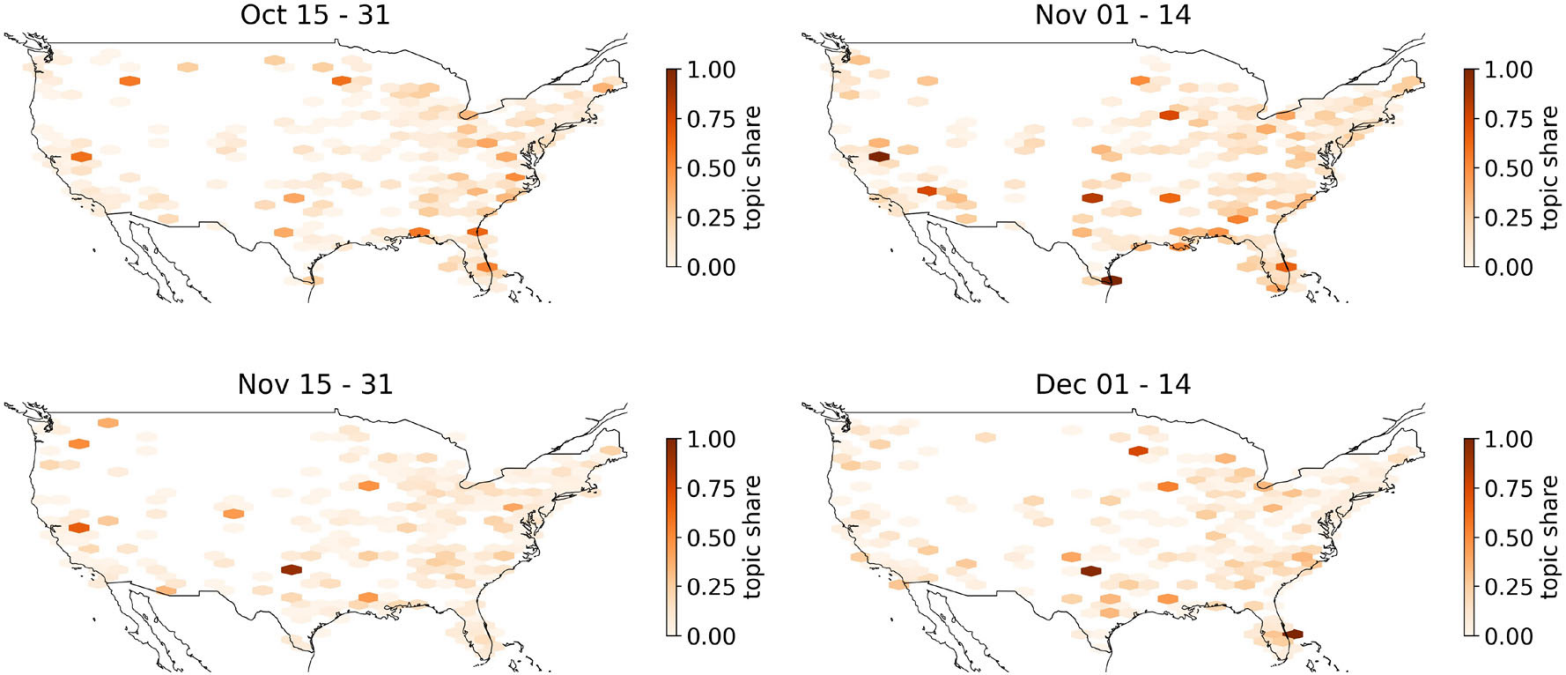
Mapplot of Covid-19-LDA topic 13—election fraud

In the following, we focus on the spatial distribution of the topics. Figure 8 displays the geo-spatial distribution of the “Election Fraud”-topic 13 in four time intervals. The map is organized into hexagons. The coloring of the hexagon represents the share of tweets of topic 13 in relation to the total number of tweets in the respective hexagon region at the indicated time period. A hexagon is displayed if at least one tweet was posted. Empty regions mean that no tweets on topic 13 were posted. While the “Election Fraud”-topic 13 is already prevalent in late October, in November the topic’s share increases. Several hexbins in rural regions display a 100% topic share of the “Election Fraud” topic. In late November and early December, we observe decreased topic prevalence across the USA, consistent with our findings from the time series plot in Figure 6. However, the topic remains highly active in some regions, as indicated by the single dark-colored hexbins.

### Topic modeling: spatiotemporal characteristics of the Conspiracy discussion

**Fig. 9:**
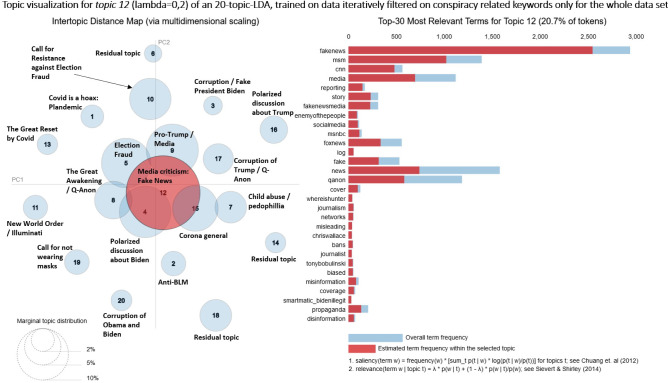
LDA visualization of total time period—only conspiracy-related tweets

Figure 9 presents the topical analysis of the LDA model trained with the small conspiracy-tweets dataset. As mentioned above, conspiracy theories consist of approximately 10% of the words from the larger Covid-19-tweets dataset. The inter-topic distance map (Figure 9) shows a similar pattern as in the large dataset (Figure 5). The majority of topics are concentrated in the center of the PCA representation, small “satellite” topics are located at the peripherals. For the Conspiracy-tweets dataset, this centralized topic structure is surprising, as we expected to see various independently discussed conspiracy theories. This is probably a side effect of the dominance of election-related conspiracies. Figure 9, however, suggests a large overlap between conspiracies. Moreover, the majority of topics is related to Donald Trump, whereas “classical” conspiracy theories are marginal.

**Fig. 10:**
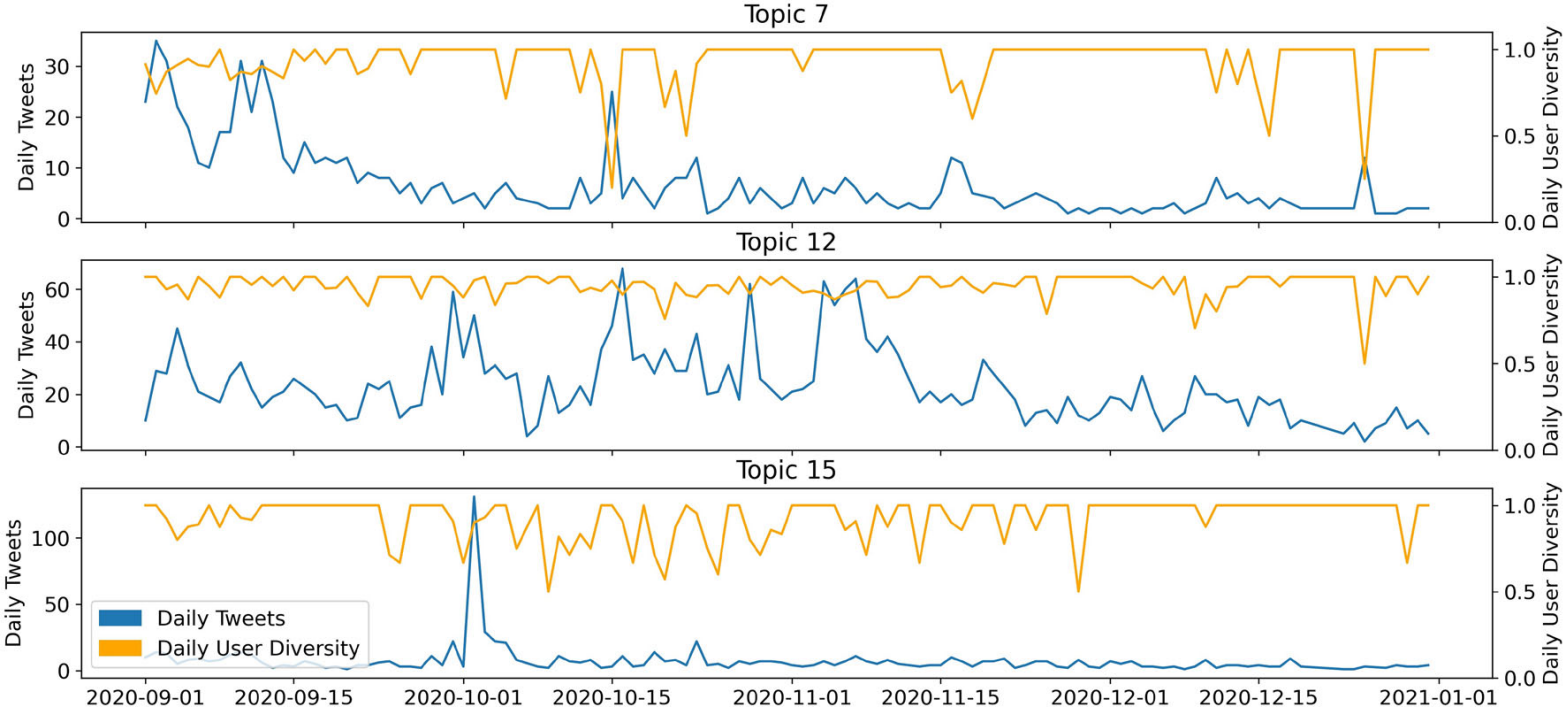
Time series of conspiracy-LDA topics 7, 12, 15

Note that an indication of the consistency of our method is provided by the reappearance of the “schadenfreude”-topic, here topic 15 (7.8% of all tokens). The associated time-series plot in Figure 10 is almost identical to that of topic 1 in the large dataset (Figure 6), only the absolute size of the associated tweets differs by a factor of 10.

**Fig. 11:**
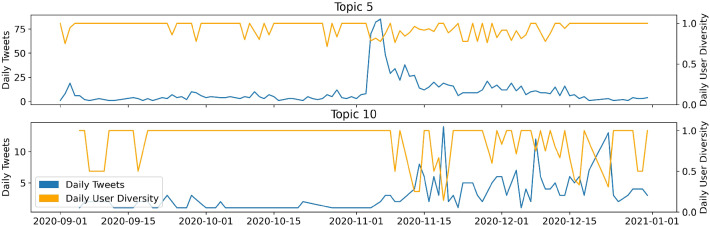
Time series of conspiracy-LDA topics 5, 10

**Fig. 12:**
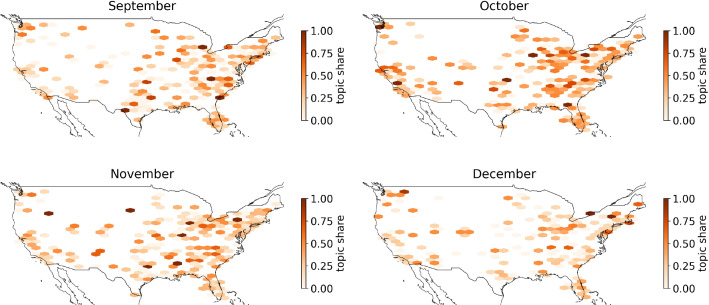
Mapplot of conspiracy-LDA topic 12—fake news

Notably, the LDA distinguishes between two different election fraud topics, namely topics 5 (6.5%) and topic 10 (9.2%). The main difference is that topic 10 includes calls for active resistance against the election results, whereas topic 5 appears more moderate. The time-series in Figure 10 characterizes topic 5 as a short-term topic with a peak on the election date (3rd of November, 2020), and a subsequent fade-out. The user diversity drops below 0.8 at this date. Before and after the peak, fluctuations in user diversity should not be over-interpreted, as the sample size is smaller here, resulting in a wiggly graph. In contrast, topic 10 is characterized by several peaks. Far fewer tweets are assigned to this topic than to topic 5. The small sample size in topic 10 also causes strong fluctuations in user diversity over the whole time period. It can be concluded that the calls for resistance on Twitter occur at certain points and are induced by a few active users. The first peak in mid-November with a sample size of thirteen tweets is caused by only two different users. This is reflected by a very low user diversity of around 0.15 (Figs. 11 and 12).

**Fig. 13:**
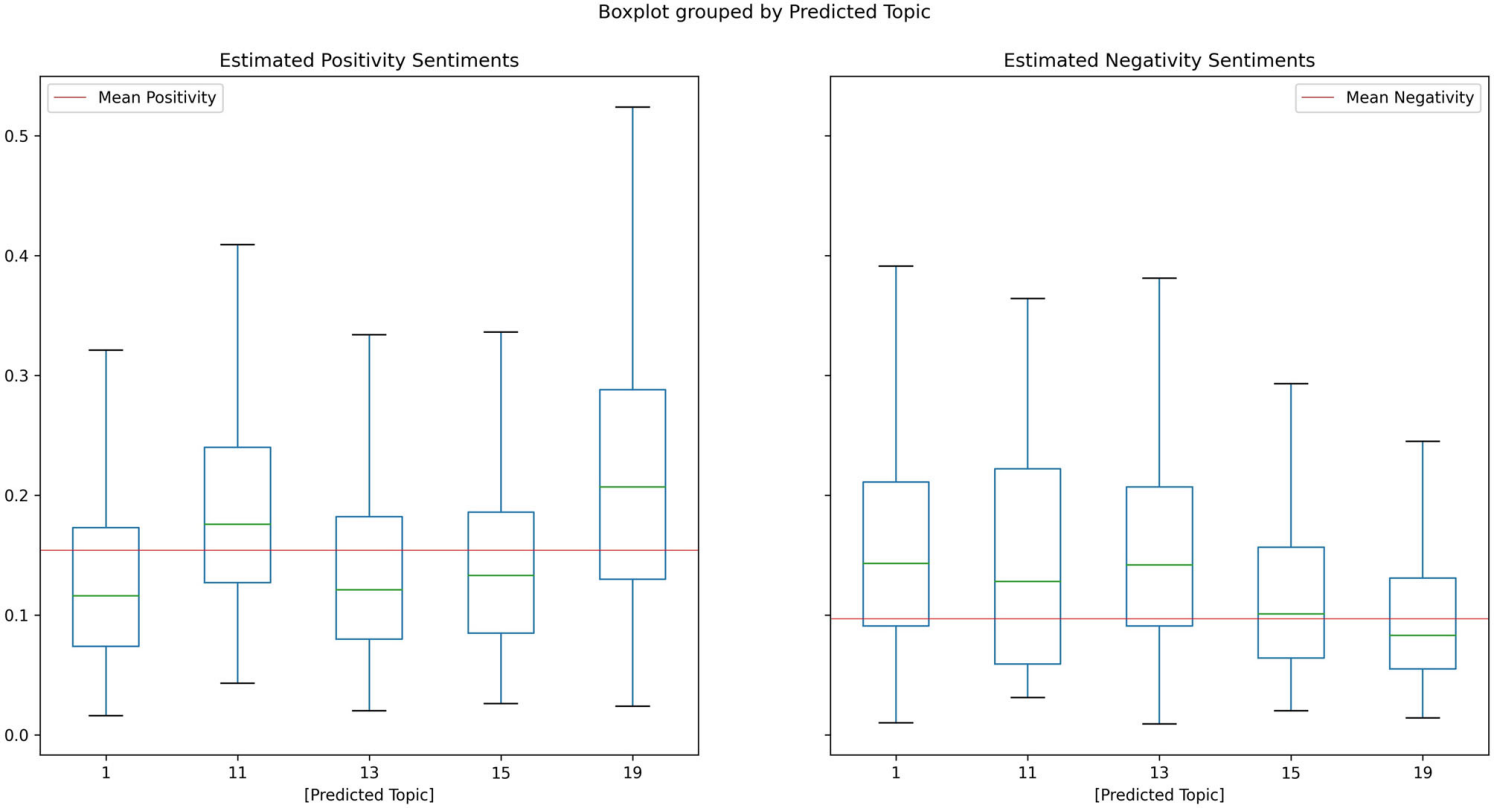
Sentiment distribution of selected conspiracy-LDA topics

The biggest topic 12 (20.7%) is about “Fake News” and contains both sides of a controversial discussion. There are mainly tweets criticizing “Main Stream Media” (MSM) appearing in topic 12. Tweets with opposing opinions can also be found, but less frequently. These tweets criticize the inflationary use of the term “Fake News.” They also claim that “MSM”-critics are brainwashed by conspiracy theories. Figure 10 shows that the topic is discussed over the entire period, with less activity after the US-Presidential Election. This is another indication that debates on Twitter, especially in relation to conspiracy theories, changed significantly as a result of the election. Figure 8 shows the spatial distribution of the “Fake News” Topic by month. We find that the topic is most prevalent from September to November, with a decline in December. While the topic is more or less prevalent in most parts of the country, we see a shift to a higher ratio of this topic to rural areas, especially in November. We suggest this trend has to do once more with the US-Presidential Election, where the “Fake News” claims go hand-in-hand with the “Election Fraud” myth.

Figure 16 in the Appendix represents the spatial distribution of two small “satellite” topics. Topic 11 (2.3%) represents a discussion of the “New World Order” conspiracy theory. This theory claims that global elites try to introduce a global world government to the detriment of the general population [9]. Topic 19 (2.3%) is an appeal against wearing (surgical) face masks. One can easily see that these topics are almost exclusively discussed in a few specific locations. For topic 11, we find a focal point hexagon in rural Texas, for topic 19 in rural Missouri. These thematically very different topics show surprising similarities in their spatial distribution: the “Texas” hexbin also reappears for topic 19 if we consider the period of the first half of December. In summary, the data seems to indicate that the same users, or locally networked user groups, are tweeting about “classical” conspiracies (Topic 11) as well as current Covid-19-related topics. It also suggests that attempts are being made by small groups, or individuals using bots, to gain influence on the discourse on Covid-19 by spreading topic-related conspiracy theories.

Topic 7 (3.8%) is located in the PCA representation (Fig. 5) somewhat apart from the central topic cluster. Child abuse is discussed under this topic. There is a lot of discussion about this topic in September with another peak in October followed by a fade-out (Fig. 10). The topic could be linked to the release of the Netflix series “Cuties,” as well as the lawsuit about the case of a young girl, which was much discussed among Twitter users under the hashtag *standwithsophie*. The clearly conspiracy-related terms *qanon* and *savethechildren* hashtags are also found in the word distribution of topic 7. This might be a strategy to mobilize Twitter users via hard-to-oppose topics such as child protection and thus increase the reach of other conspiracy theories as well.

### Sentiment analysis of topics

This Section evaluates if conspiracy-related topics differ sentiment-wise from other topics and how they are spatially distributed.

**Fig. 14:**
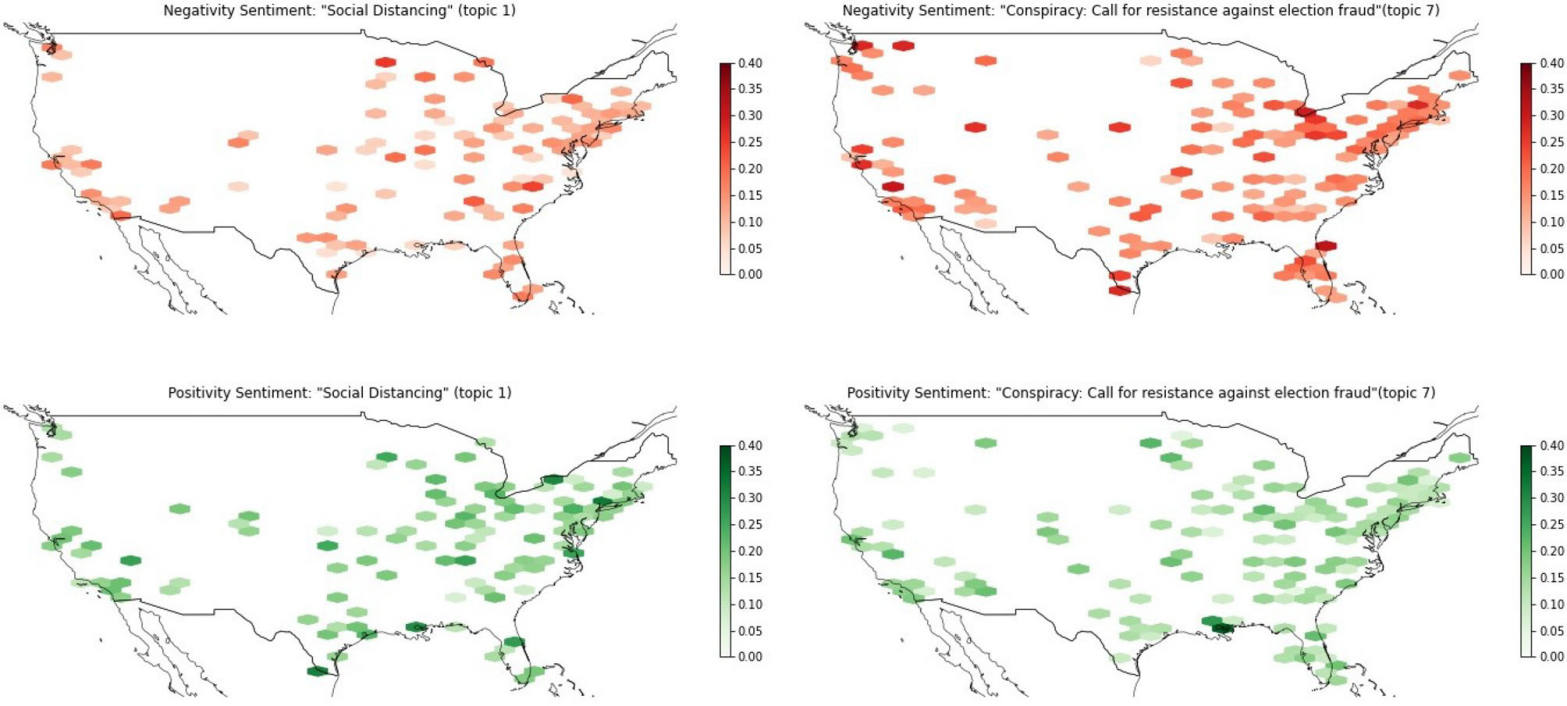
Sentiment maps of an eight-topic-LDA, trained on data iteratively filtered on Covid-19 and conspiracy-related keywords for December 2020: upper left: “Negativity Sentiment: ‘Social Distancing’ (topic 1),” upper right: “Negativity Sentiment: ‘Conspiracy: Call for resistance against election fraud’(topic 7),” lower left: “Positivity Sentiment: ‘Social Distancing’ (topic 1),” lower right: “Positivity Sentiment: ‘Conspiracy: Call for resistance against election fraud’(topic 7)”

**Fig. 15:**
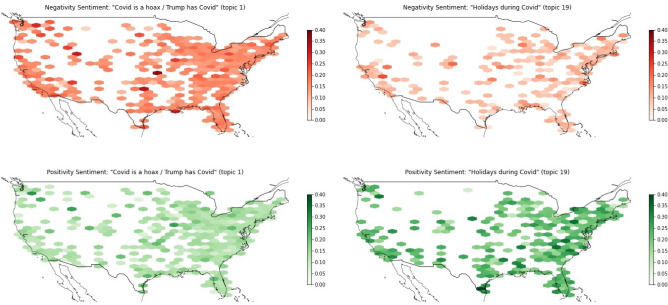
Sentiment maps of a twenty-topic-LDA, trained on data iteratively filtered on Covid-19 and conspiracy-related keywords for September–December 2020: upper left: ’Negativity Sentiment: “Covid is a hoax / Trump has Covid” (topic 1)’, upper right: ’Negativity Sentiment: “Holidays during Covid” (topic 19), lower left: ’Positivity Sentiment: “Covid is a hoax / Trump has Covid” (topic 1)’, lower right: ’Positivity Sentiment: “Holidays during Covid” (topic 19)’

Figure 13 describes the sentiment distribution of the conspiracy-related topics 11, 13 and 15, as well as two non-conspiracy benchmark topics 1 and 19. Topic 1 is about Trump’s Covid-19 infection for which we expect increased negative sentiments. Topic 19 is concerned with holidays in Covid-19 times and is expected to be classified positively. Boxplots are displayed to visualize sentiments distributions by topics. The left side of the Figure displays the positive, the right side the negative sentiment distributions. The red bar indicates the sentiment mean of all Covid-19-related tweets, excluding residual and conspiracy topics. Our expectations are confirmed by the data: The majority of topic 1 tweets lays below the positivity mean and above the negativity mean. For topic 19, the opposite pattern is observed: holiday-tweets score higher than average on positivity and slightly lower than average on negativity. However, its total negativity mean is close to the center of the inter-quartile range, indicating that its negativity does not considerably differ from overall negativity. The conspiracy topics display a mixed pattern. Their negativity sentiments are slightly larger than usual, with only topic 13 being placed well above the mean line. Topics 13 and 15 show a slightly lower positivity whereas topic 11 is somewhat more positive than the rest.

Figures 14 and 15 display a sentiment comparison. The first example in Figure 14 contrasts the topics “Social distancing” and “Call for resistance against election fraud” form the eight-topic-LDA model for December 2020. The Covid-19-related topic “Social distancing” contains tweets that are more positive and also tweets that are less negative in comparison to the “Call for resistance against election fraud” topic. Also for the second example in Figure 15, we see that even though both topics are Covid-19-related, topic “Covid is a hoax / Trump has Covid” contains many tweets that are less positive and many tweets that are more negative than the “Holidays during Covid” topic. We find that the main negativity clusters can be found in parts of the US further away from the coasts for the Covid-19-hoax topic.

In conclusion, our findings suggest that tweets with a high prevalence of conspiracy-related topics tend to be more negative in terms of their sentiment than tweets with a high prevalence of non-conspiracy-related topics. Nevertheless, this hypothesis could be further validated by additional research conducted on a larger dataset than ours.

## Conclusion

**Fig. 17:**
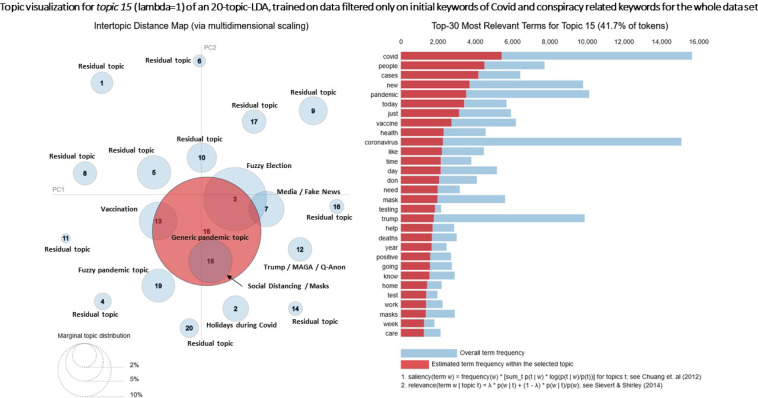
LDA visualization of topics if filtered only on initial keywords

**Fig. 18:**
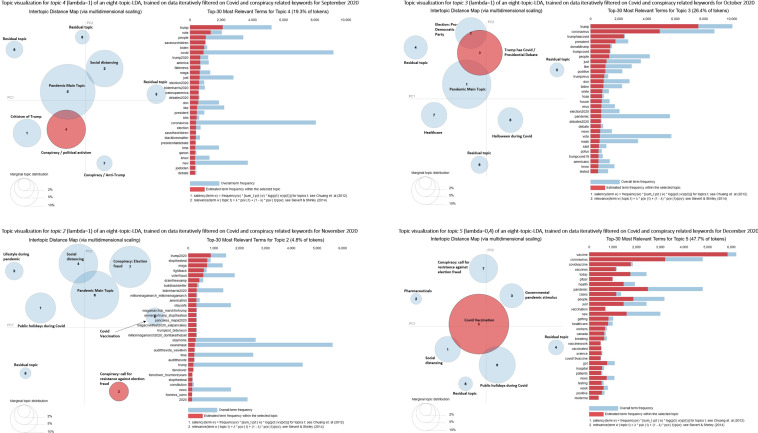
LDA visualizations by month of Covid-19 and conspiracy-related tweets

**Fig. 19:**
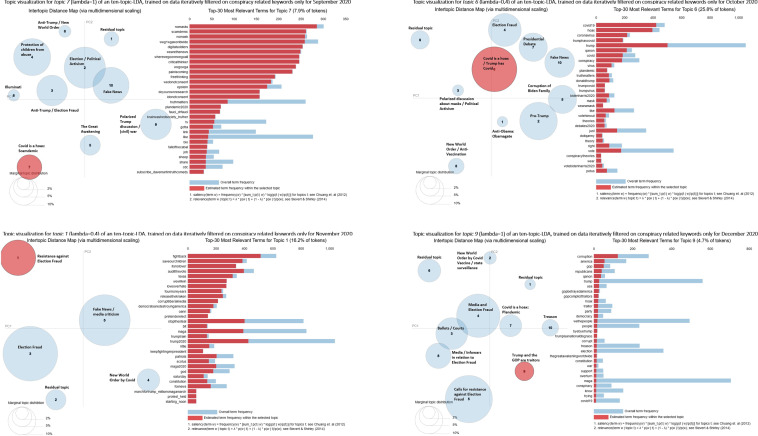
LDA visualizations by month of only conspiracy-related tweets

The results of the Iterative Filtering show a substantially improved coherence within the generated topics. The topic models perform well in terms of coherence and produce only some residual topics which can be clearly differentiated from the topics of interest. Most of the non-related tweets are grouped in “residual topics.” This is clearly different from the results produced by only using the initial filter keywords, in Figure 17 in the Appendix, where the amount and ratio of the residual topics is much higher, while the coherence of the non-residual topics is lower. When applying Iterative Filtering, the controversial topics, which are often smaller in size (with a smaller number of tweets), especially benefited from the removal of the non-related words that we put on the “blacklist” dictionary.

In general, we saw that many conspiracy-related topics are marginal in comparison with other mainly discussed topics, like the US-Presidential Elections or general discussions about Covid-19. The main conspiracy theories present within the public discourse are related to the general focus of the discussion: they are dominated by “Election Fraud” in November and December and by the “Covid-19-hoax” theory in general. The conspiracy-related keywords are often grouped together with Trump-related words and words related to his presidential campaign. This implies that users who are utilizing conspiracy-related hashtags are often discussing Trump-related topics. Our framework can be utilized as a basis for further analyses of the topics of interest as we have shown with our brief geo-spatial Sentiment Analysis. We find that nation-wide conspiracy-related topics tend to be less positive and more negative sentiment-wise in comparison with topics focusing on Covid-19-related discussions in general.

With our Iterative Filtering method, we are able to generate Twitter datasets that can be very well processed by LDA Topic Models. Thereby, we are able to gain insights into discussions of interest on Twitter and similar platforms: on the one hand, our method allows us to zoom into small sub-topics (like conspiracy-related topics) while also providing an overview of the broader context of the discussion (like election and Covid-19-related topics) over time. Hence, sub-topics, which are in general hard to identify due to small sample sizes in an overall larger dataset, are much more accessible within our framework. They also become much more coherent and therefore interpretable. Thereby we provide researchers a tool for further research on marginalized topics.

## Figures and Tables

**Fig. 16:**
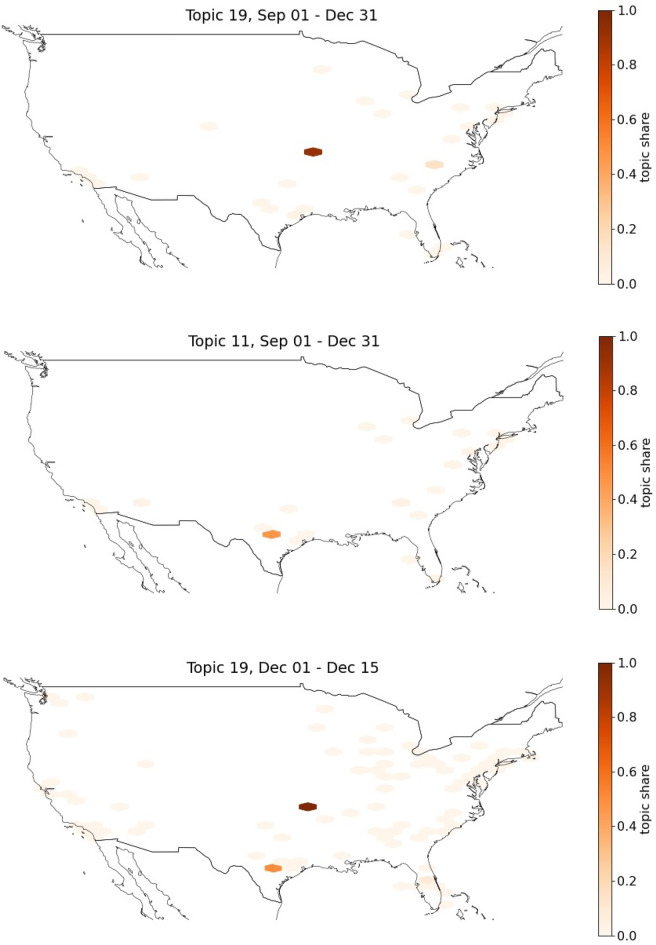
Mapplot of conspiracy-LDA topics 11, 19

## Appendix

See Figs. 16, 17, 18 and 19

## Footnotes

1 Note that methods that use web-scraping to generate labeled data could be a potential remedy to generate labeled out-of-domain data to train a classifier [27–29]. However, finding conspiracy-related out-of-domain data for tweets via web-scraping is very challenging and more time intensive than our approach.

2 Python version 2.8.1 used here.

3 Gensim version 3.8.3 used here.

4 In November 2017, Twitter doubled the available character space from 140 to 280 characters.

5 The total maximum amount of tweets that can be streamed is theoretically defined in relation to the number of GET requests per 15 minutes (Standard Twitter API v1.1) but this is practically bound by the computational power and internet connection strength of the machine streaming the tweets. More detailed information on the rate limit can be found under https://developer.twitter.com/en/docs/twitter-api/v1/rate-limits. More info about the implementation of streaming tweets can be found under https://docs.tweepy.org/en/stable/streaming.html.
